# *CasEcR* and *CasMIH* Genes in the Blue Crab, *Callinectes sapidus*: A Temporal Evaluation and Melatonin Effects

**DOI:** 10.3389/fphys.2022.903060

**Published:** 2022-06-21

**Authors:** Daniela Dantas David, Leonardo Vinícius Monteiro de Assis, Maria Nathalia Moraes, Flávia Pinheiro Zanotto, Ana Maria de Lauro Castrucci

**Affiliations:** ^1^ Laboratory of Comparative Physiology of Pigmentation, Department of Physiology, Institute of Biosciences, University of São Paulo, São Paulo, Brazil; ^2^ Center of Brain, Behavior and Metabolism, Institute of Neurobiology, Lübeck University, Lübeck, Germany; ^3^ Laboratory of Neurobiology, Department of Physiology and Biophysics, Institute of Biomedical Sciences, University of São Paulo, São Paulo, Brazil; ^4^ Department of Physiology, Institute of Biosciences, University of São Paulo, São Paulo, Brazil; ^5^ Department of Biology, University of Virginia, Charlottesville, United States

**Keywords:** eyestalk, hepatopancreas, molt cycle, ecdysteroids, molt-inhibiting hormone

## Abstract

Environmental cues synchronize endogenous rhythms of many physiological processes such as hormone synthesis and secretion. Little is known about the diurnal pattern of hormones and gene expression of the *Callinectes sapidus* molt cycle. We aimed to investigate in the eyestalk and hepatopancreas of premolt and intermolt *C. sapidus* the following parameters: 1) the diurnal expression of the ecdysteroid receptor *CasEcR* isoforms, and the molt inhibiting hormone *CasMIH;* 2) the diurnal hemolymph ecdysteroid and melatonin levels; and 3) melatonin effects on the transcripts of the above-mentioned genes in intermolt *C. sapidus*. Ecdysteroid levels were higher in the premolt than the intermolt animals at all time points evaluated (ZTs). Premolt crabs displayed a variation of ecdysteroid concentration between time points, with a reduction at ZT17. No difference in the melatonin level was seen in either molt stage or between stages. In the eyestalk of intermolt animals, *CasEcR* expression oscillated, with a peak at ZT9, and premolt crabs have a reduction at ZT9; *CasMIH* transcripts did not vary along 24 h in either molt stage. Moreover, the evaluated eyestalk genes were more expressed at ZT9 in the intermolt than the premolt crabs. In the hepatopancreas, *CasEcR* expression showed a peak at ZT9 in premolt crabs. Exogenous melatonin (10^−7^ mol/animal) reduced the expression of both genes in the eyestalk at ZT17. In the hepatopancreas, melatonin markedly increased the expression of the *CasEcR* gene at ZT9. Taken altogether, our results are pioneer in demonstrating the daily oscillation of gene expression associated to molt cycle stages, as well as the daily ecdysteroid and melatonin levels and the remarkable influence of melatonin on the molt cycle of *C. sapidus*.

## 1 Introduction

The crustacean molt cycle is a complex process comprising behavioral, morphological, and physiological events that ultimately promote differentiation and growth (Lipcius and Herrnkind, 1982; Chang, 1995). The crustacean molt cycle may be staged in four phases: premolt (D), ecdysis (E), post-molt (A-B), and intermolt (C) (or anecdysis) (Spindler et al., 1974; Kuballa and Elizur, 2007). Every molt cycle stage is subdivided into several sub-stages. These sub-stages are comprised of morphological and physiological changes as a consequence of hormone fluctuations found in each stage (Loeb, 1993; Chang and Mykles, 2011).

The positive regulation of the cycle is mainly exerted by cholesterol-derived hormones, the ecdysteroids, which are synthesized and released by the Y-organ as inactive forms (Spaziani and Wang, 1993). The ecdysteroids are converted to molt-inducing factors in the target organs (Böcking et al., 1995; Spaziani et al., 1997), thus stimulating tissue-specific responses that culminate with ecdysis. The ecdysteroids bind to a nuclear receptor (*EcR*) that forms a heterodimer with the retinoic acid receptor (*RXR*) or ultraspiracle protein (*USP*), to evoke their responses (Chen et al., 2017). In *C. sapidus CasEcR* and *CasRXR* co-express in the eyestalks and Y-organ, but with different levels, suggesting response and sensitivity differences to ecdysteroids (Techa and Chung, 2013). In the insect *Rhodnius prolixus*, the ecdysteroid receptor oscillates in some of the hormone targets exhibiting a robust diurnal rhythm synchronized with the circulating levels of ecdysteroid, and daily migrating from the cytoplasm to the nucleus (Vafopoulou and Steel, 2006). In general, ecdysteroid concentration is low during the intermolt, peaks in the premolt, followed by a decrease just before the ecdysis (Chung, 2010).

Ecdysteroid release as well as its action in peripheral tissues are regulated by X-organ hormones localized in the crustacean eyestalk (Asazuma et al., 2009). The X-organ is responsible for producing and releasing many peptide hormones like the pigment dispersing and concentrating hormones (PDH and PCH, respectively), and the hyperglycemic hormone (CHH) superfamily (Hopkins, 2012; Webster et al., 2012; Katayama, 2016). The molt inhibiting hormone (MIH) is a representative peptide hormone of the CHH-type II family (Nakatsuji et al., 2009); its receptor is a membrane guanylyl cyclase, also identified in *Callinectes sapidus* (Zeng et al., 2008). MIH inhibits ecdysteroid production and secretion, through receptor binding on Y-organ (Spaziani et al., 1999). Hemolymph levels of the two hormones, ecdysteroids and MIH, vary in anti-phase throughout the cycle, i.e., in the premolt, the ecdysteroid concentration is high, whereas MIH remains high during intermolt and post-molt (Lee et al., 1998; Nakatsuji et al., 2000; Nakatsuji and Sonobe, 2004).

Another hormone, methyl farnesoate, is considered an accessory hormone of the molt induction, showing higher concentrations in the premolt stage (Nagaraju et al., 2006; Nagaraju, 2007; 2011); it is produced by the mandibular organ, increases ecdysteroid concentration in the Y-organ cells, and accelerates the molt cycle (Reddy et al., 2004).

In addition to the endocrine organs classically involved in the molt cycle, like the X-organ and the Y-organ, the hepatopancreas (midgut gland) also participates in the molt cycle. The hepatopancreas is an essential metabolic tissue responsible for the storage and distribution of nutrients, whose demands change throughout the cycle (Lipcius and Herrnkind, 1982; Huang et al., 2015). The proteomic analysis of *Scylla paramamosain* hepatopancreas demonstrated that 193 proteins responsible for exoskeleton and cuticle reconstruction, energy reserves, immune responses, and metabolism were altered between the molt stages (Liu et al., 2021). During the *Eriocheir sinensis* vitellogenesis, the hepatopancreas expresses four *EcR* isoforms with the same profile, which play a more important role during the earlier ovarian development stages (Su et al., 2020). Furthermore, the organ produces vitellogenin that is stimulated by MIH during the process of ovarian maturation in *C. sapidus* (Zmora et al., 2009; Luo et al., 2015).

Environmental cues, named *zeitgebers* (time givers in the German language), comprise physical factors such as light, temperature, salinity, and tides among others, which may have been imposed on the organisms since the species origin. These factors affect the physiological processes by synchronizing their endogenous rhythms with each other and with the environment (Stoner et al., 2013; Espinosa-Chaurand et al., 2017). This feature allows biological processes to occur in an appropriate order, thereby preventing concurrent activation of potentially incompatible mechanisms.

Among a variety of biological processes, endocrine signaling is one of the most integrative diurnal systems. In this sense, melatonin, the messenger of the darkness, emerges as an important hormone that contributes to the whole-body temporal regulation in vertebrates (Reiter et al., 2009; Markus and Ferreira, 2011; Dardente, 2012; Cipolla-Neto et al., 2014; Cipolla-Neto and Amaral, 2018). Melatonin is a ubiquitous and amphiphilic molecule derived from the amino acid tryptophan, which is converted to the bioactive hormone due to the action of the enzymes aryl-alkyl aminotransferase (AANAT) and hydroxymethyl transferase (ASMT) (Axelrod and Weissbach, 1960; Vivien-Roels and Pévet, 1993; Tan et al., 2016). It is widely accepted that melatonin oscillates in a circadian pattern in all vertebrates studied to date, peaking in the dark phase, because the biosynthesis limiting enzyme—AANAT—is inhibited by light through clock-regulated signaling (Wurtman et al., 1963; Reiter et al., 2014).

In Crustaceans, on the other hand, there is no consensus as the time of the day at which melatonin peaks in the various species as well as if this hormone displays an oscillatory pattern (Agapito et al., 1995; Tilden et al., 1997; Maciel et al., 2008; Markowska et al., 2009; Sainath et al., 2013; Han et al., 2018). As to melatonin actions in crustaceans, it has already been demonstrated that the indoleamine plays a role in limb regeneration (Tilden et al., 1997), ecdysteroid production (Sainath S. B. and Reddy P. S., 2010; Girish et al., 2015), antioxidant defense (Maciel et al., 2010; Geihs et al., 2016; She et al., 2019), color change (Nery et al., 1999), locomotor activity (Geihs et al., 2010), and hyperglycemia (Sainath SB. and Reddy PS., 2010; Maciel et al., 2014; Yang et al., 2018), among others. Many melatonin functions in crustaceans are related to the molt cycle, as shown by the increased levels of ecdysteroids in melatonin-treated Y-organ cells (Girish et al., 2015).

Little is known about the diurnal oscillation of molting hormones, and their role in the daily regulation of the molt cycle. In this study, we aimed to evaluate whether the gene expression of the ecdysteroid receptor (*CasEcR*) and the molt-inhibiting hormone (*CasMIH*) and the secretion of ecdysteroids and melatonin displayed a diurnal profile in intermolt and premolt animals. These molt stages were selected because they show the most striking hormone anti-phase peaks, which may be related to the parameters we chose to measure. Here we report a diurnal pattern of molt cycle-related genes and ecdysteroid and melatonin levels, suggesting melatonin as a positive modulator of the molt cycle in *C. sapidus*.

## 2 Material and Methods

### 2.1 Animals

**FIGURE 1:**
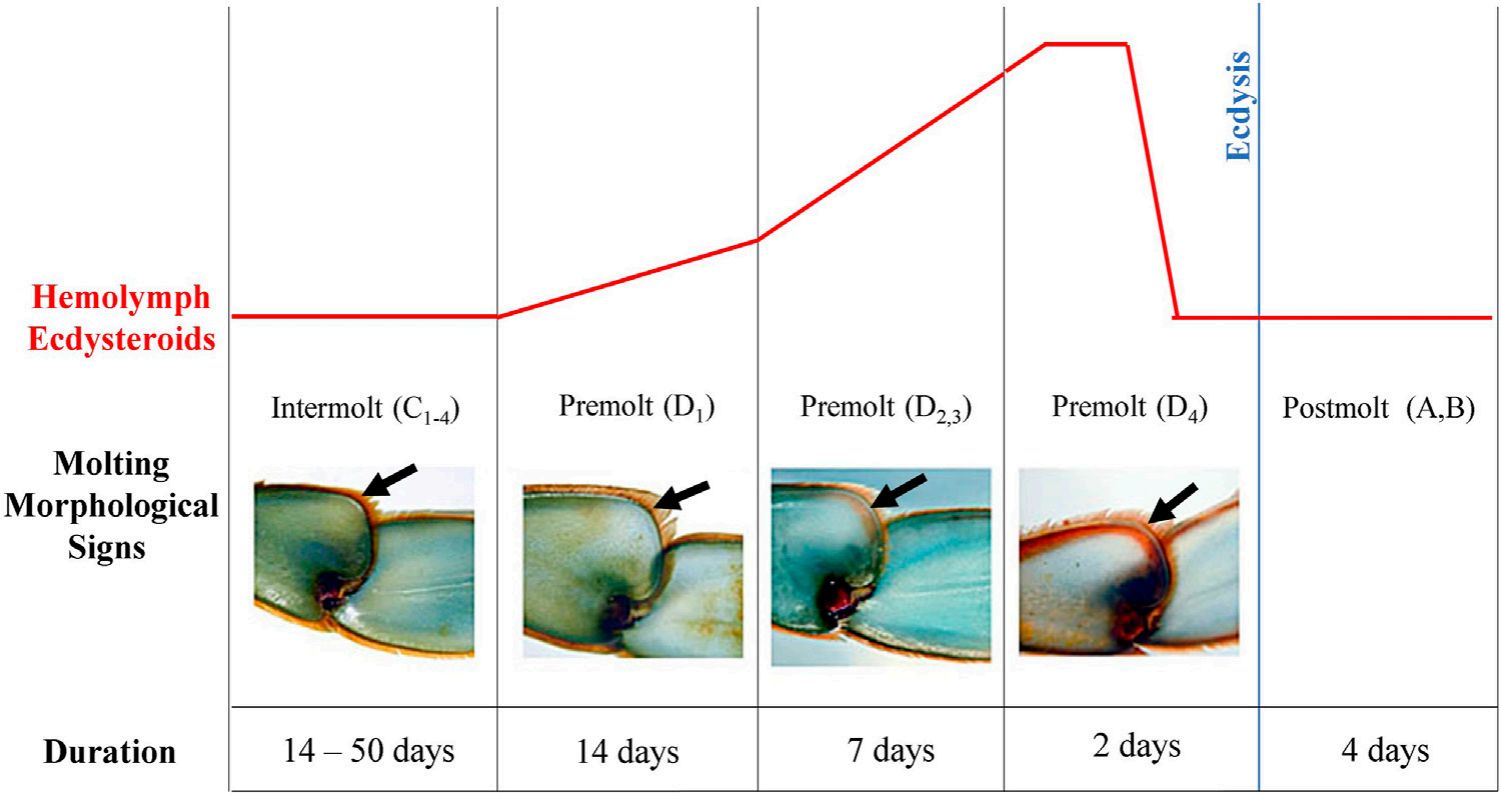
Illustration of molt cycle of the blue crab, *Callinectes sapidus*. The diagram shows the hemolymph ecdysteroid titer pattern (upper panel), morphological signals of the leg (black arrows), and duration of each stage during the molt cycle (bottom panel). Modified from Blue Crab. Info (https://www.bluecrab.info/redsign.htm).

Males and females (average weight: 55.64 ± 10.7 g, and average carapace width: 9.5 ± 0.58 cm) of *Callinectes sapidus* were purchased from fishermen in Iguape city, state of São Paulo, Brazil, between March and May of 2017. The animals were transported to the University of São Paulo and kept for at least 3 days in an open water system with constant aeration, 10 ppm salinity, 22°C, under light/dark cycles of 12:12 LD (white light, 420–750 nm, 400 lux, 58 μW/cm^2^), lights on 7 a.m. (ZT0) and off 7 p.m. (ZT12) (*zeitgeber* time - ZT). For all experiments, the animals’ molt cycle stages were determined according to the absence (intermolt) or presence (premolt) of the line on the 5th pereiopod (Figure 1) and the ecdysteroid levels (intermolt = <60 ng/ml; premolt = >60 ng/ml, according to Techa and Chung, 2013 and Roegner et al., 2019). Both sexes were used since there were no sex-related differences in the chosen outputs. Animal maintenance and experimentation were authorized by the Brazilian Ministry of the Environment (license SISBIO number 67295–3).

### 2.2 Experimental Design

#### 2.2.1 Hormone Concentrations and Gene Transcript Levels in Premolt and Intermolt *C. sapidus*

**FIGURE 2:**
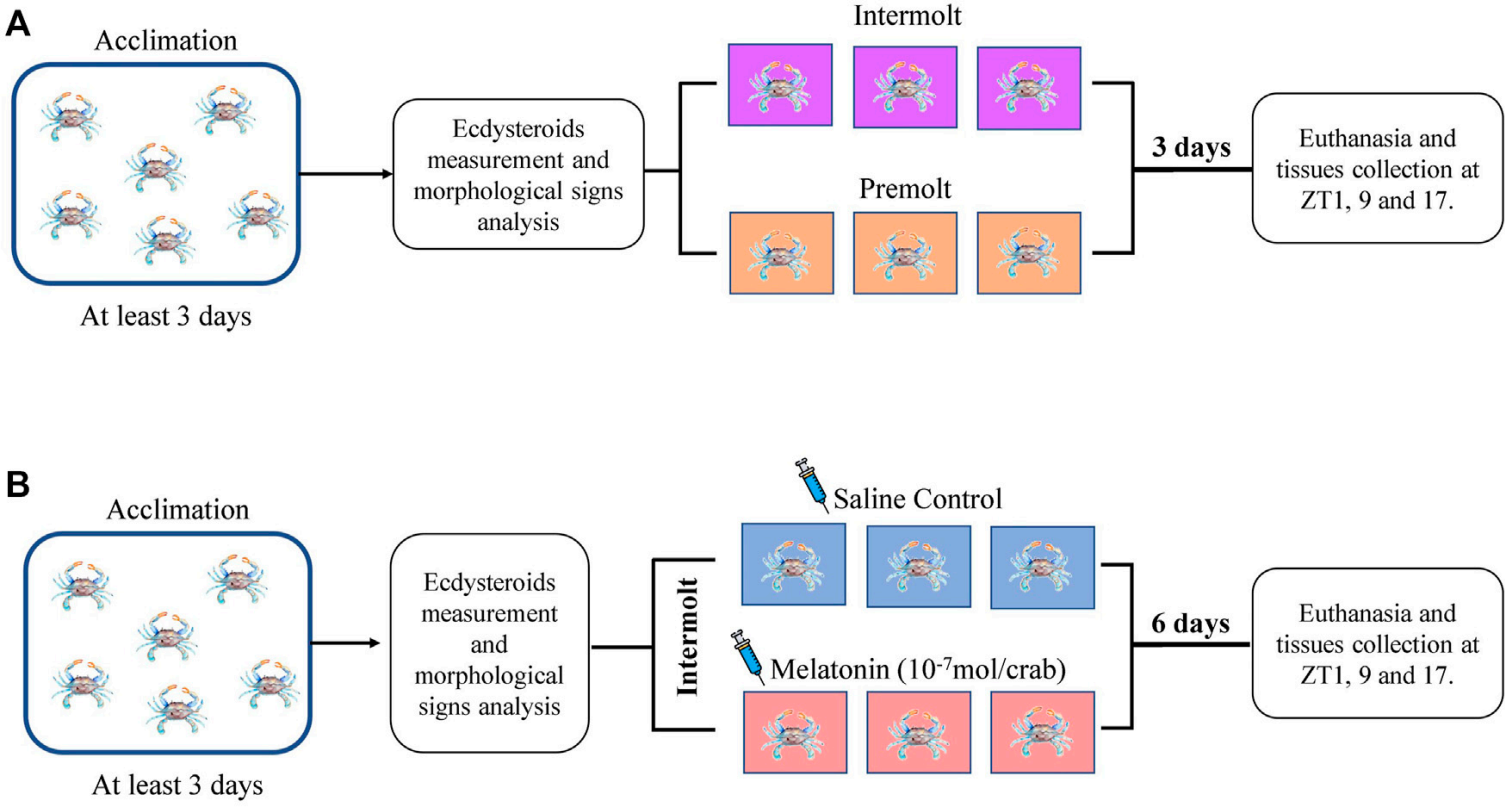
Experimental designs. **(A)** Timeline of the experiment to determine hemolymph ecdysteroids and gene expression of the intermolt and premolt crabs. **(B)** Timeline of the experiment to determine melatonin-injection effect on the hemolymph ecdysteroids and gene expression of the intermolt crabs.

Premolt and intermolt animals previously identified and acclimated for at least 3 days under the conditions mentioned above were single housed in a closed circulation system for three more days. Samples were obtained every 8 h, at ZT1 (8 a.m.), ZT9 (4 p.m.), and ZT17 (midnight) (Figure 2A). Animals were cryo-anesthetized, the hemolymph (300 µL) was collected, centrifuged at 4,000 x g, at 4°C for 10 min, and the supernatant was stored at −80°C for later melatonin and ecdysteroid measurements; hepatopancreas and eyestalk were excised and stored at −80°C for qPCR analysis.

#### 2.2.2 Melatonin Effects on the Hormone Concentrations and Gene Transcript Levels in Intermolt *C. sapidus*

Melatonin (Sigma-Aldrich, St. Louis, MO, United States) was dissolved in 100% ethanol for a stock solution of 10^−3^ M, which was further diluted in 100 µL of Pantin solution (400 mM NaCl; 10 mM KCl; 27 mM anhydrous Na_2_SO_4_; 2.4 mM NaHCO_3_; 52.13 mM MgCl_2_.6H_2_O; 7.6 mM anhydrous CaCl_2_), pH 7.6, to obtain 10^−7^ mol/100 µL (Girish et al., 2015). This volume was daily injected at 1 p.m. (ZT6), into the arthrodial membrane of the 5th pereopod of intermolt crabs for 7 days. Control animals received the same volume of the eluent containing 1% ethanol. After that, the animals were processed as described above for hemolymph and organs collection (Figure 2B).

### 2.3 Measurement of Hormone Concentrations

Hemolymph ecdysteroid levels were determined by Enzyme-Linked Immunosorbent Assay (ELISA) adapted from McKinney et al. (2017). Melatonin quantification was also determined by ELISA according to the kit manufacturer’s instruction (IBL International, Flughafenstr, Hamburg, Germany).

### 2.4 Total Ribonucleic Acid Extraction and Reverse Transcriptase Reaction (Polymerase Chain Reaction)

The eyestalk and hepatopancreas were homogenized in Trizol (Ambion, Carlsbad, CA, United States), and after the extraction of total RNA, the samples were treated with DNAse I (turbo-DNase, Life Technologies, Carlsbad, CA, United States) following the manufacturer’s instructions. RNA concentration was determined in a Nanodrop spectrophotometer (Wilmington, DE, United States), and 1 μg of RNA was reverse transcribed in a reaction containing random hexamer primers, Superscript III (Life Technologies, Carlsbad, CA, United States), and other reagents, according to the manufacturer’s instructions.

### 2.5 Quantitative Polymerase Chain Reaction

Specific primers for the molt inhibiting hormone (*CasMIH*) and ecdysteroid receptor isoform (*CasEcR*) genes and ribosomal RPL12 (*CasRPL12*, utilized as normalizer), based on the GenBank sequences (http://www.ncbi.nlm.nih.gov/genbank), were designed with PrimerBlast (Table 1) and synthesized by IDT (Coralville, IA, United States). The single gene PCR reactions contained 300 nM of each primer, KAPA SYBR^®^ Fast qPCR Mix 2x (KapaBiosystems, Wilmington, MA, United States), and DNase/RNase free water (Ambion, Carlsbad, CA, EUA). The assay was performed in the iQ5 thermocycler (Bio-Rad Laboratories, Hercules, CA, United States) as follows: 10 min at 95°C, followed by 45 cycles of 15 s at 95°C, 1 min at 60°C, and then 80 cycles of 10 s at 55°C, with a gradual increase of 0.5°C (melting curve to validate the specificity of the primers). For each gene, two replicates of each sample were used. Negative controls were routinely included with no template.

**TABLE 1:** Primers’ sequences and access numbers for the qPCR assays.

Templates	Sequences
***CasMIH***	For.:5′-CAGCTTACAAGAGCACCGGA-3′
KJ813010.1	Rev.: 5′-TTT​CTG​ACT​GAC​CGT​TGC​GT-3′
***CasECR***	
HQ630857.1	For.: 5′-CAC​GTG​TGA​CAG​TCA​GTG​GA-3′
HQ630859.1	Rev.: 5′-ACC​AGA​GCC​CAA​CAC​AAA​CA-3′
HQ630858.1	
JQ771939.1	
***CasRPL12***	For.: 5′-AAT​CGC​AGT​TCA​TCC​TCC​AC-3′
Yednock et al. (2015)	Rev.: 5′-GAG​GCA​TGG​TGC​TGA​ATT​TG-3′

### 2.6 Statistical Analysis

The mRNA levels were calculated according to the 2^-∆∆CT^ method (Livak and Schimittgen, 2001). The CT (amplification cycle) of each qPCR reaction was determined where the threshold crosses the geometrical portion of the amplification curves. ∆CT was found by subtracting *CasRPL12* CT from each gene CT. The minimal mean value was then subtracted from each ∆CT sample value to obtain ∆∆CT, placed as the negative exponential in base 2.

The optical density (OD) values in the ELISA assays were interpolated in a 4 parameters standard curve for melatonin and semilog line standard curve for ecdysteroid, to obtain the concentration values which were expressed in pg/mL and ng/mL, respectively.

Outliers of the gene expression and hormone levels were identified using the ROUT method with Q = 10%, the Shapiro-Wilk test was used to check the normality, and the data were compared by Two-way ANOVA. To evaluate the influence of variables or the interaction between them, the *F* value was analyzed [*F* (DFn, DFd), *p* < 0.05], followed by Bonferroni’s post-test to compare the time points within a group, or the same time point among groups. Results are shown as median, quantiles, maximum, and minimum expression values of genes of interest, and *p* < 0.05 was established to reject the null hypothesis. All analyses were done in GraphPad Prism 8.0.

## 3 Results

### 3.1 Circulating Hormone Levels

**FIGURE 3:**
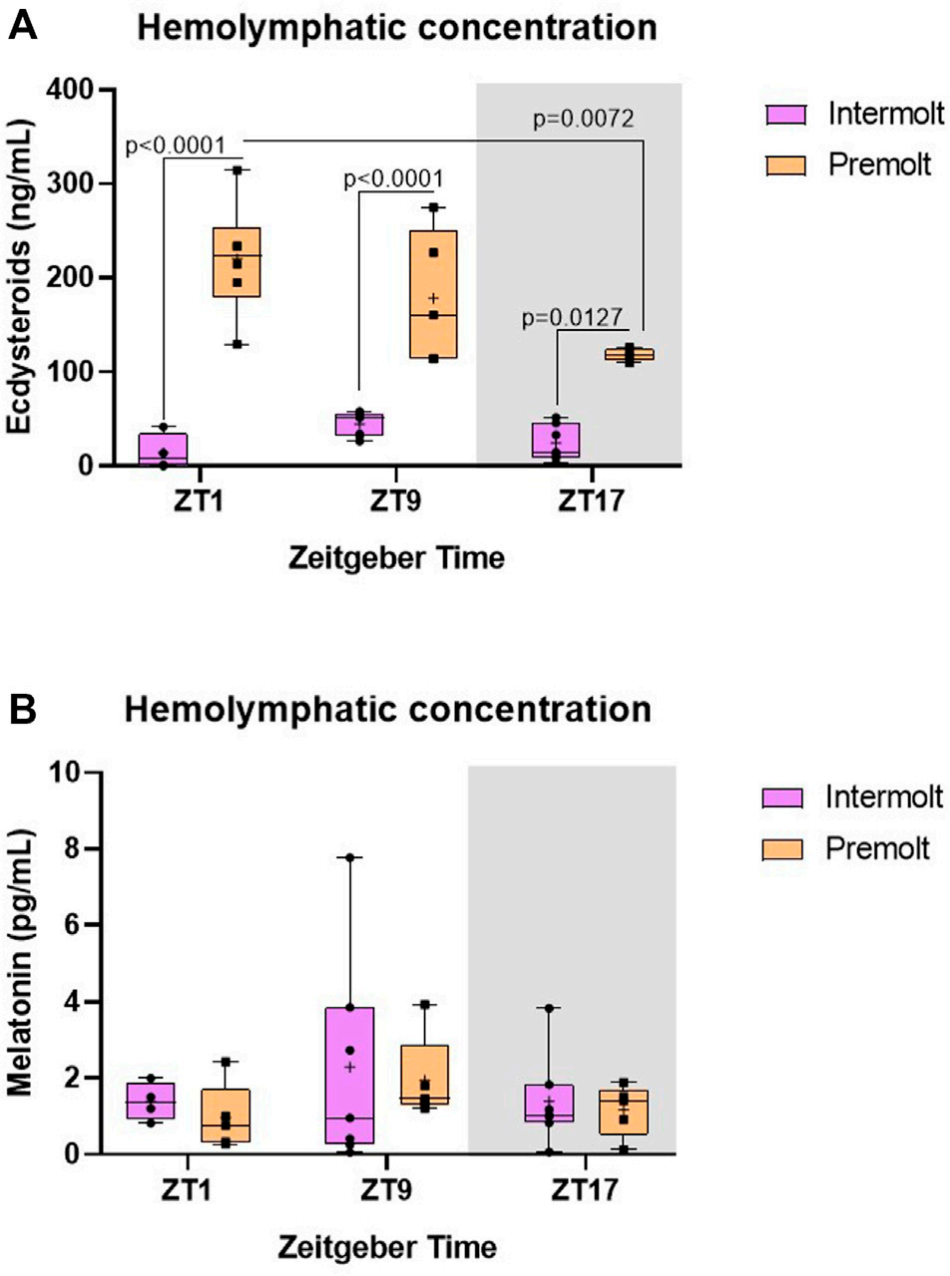
Concentration of ecdysteroids and melatonin in *C. sapidus* hemolymph. **(A)** Ecdysteroids (*n* = 4–7) and **(B)** melatonin (*n* = 4–7) were extracted 1, 9 and 17 h after lights on. The values are expressed as median, quantiles, maximum, and minimum concentration. *p* values refer to the differences between molt stages, or temporal points within a single molt group, as determined by Two-way ANOVA, followed by Bonferroni post-test. In this and the following figures, the gray rectangle represents the scotophase, and n is the animal number.

Aiming to determine if ecdysteroid levels display a rhythm along the day, hemolymph of intermolt and premolt *C. sapidus* was collected and analyzed at three-time points (ZTs). We found a significant interaction between factors (molt stage and time) on the ecdysteroid levels (Table 2). As expected, ecdysteroids showed remarkably higher levels in premolt than intermolt animals, at all evaluated time points. Additionally, in the premolt crabs, the ecdysteroids showed a decrease at ZT17 in comparison to ZT1 (*p* = 0.0072) (Figure 3A).

**TABLE 2:** ANOVA parameters related to gene expression and hormone concentrations.

		Time		Molt stage		Interaction	
		F (DFn,DFd)	*p* Value	F (DFn,DFd)	*p* Value	F (DFn,DFd)	*p* Value
Ecdysteroids	Hemolymph	(2, 27) = 4.037	***p* = 0.292**	(1, 27) = 102.5	***p* < 0.0001**	(2, 27) = 5.020	***p* = 0.0140**
Melatonin		(2, 27) = 1.202	*p* = 0.3161	(1, 27) = 0.3474	*p* = 0.5605	(2, 27) = 0.009	*p* = 0.9901
*CasMIH*	Eyestalk	(2, 31) = 0.9044	*p* = 0.4152	(1, 31) = 13.41	***p* = 0.0009**	(2, 31) = 0.4307	*p* = 0.6539
*CasECRI*	Eyestalk	(2, 25) = 0.8656	*p* = 0.4330	(1, 25) = 0.0758	*p* = 0.7853	(2, 25) = 8.296	***p* = 0.0017**
	Hepatopancreas	(2, 17) = 0.5085	*p* = 0.6102	(1, 17) = 6.217	***p* = 0.0233**	(2, 17) = 2.470	*p* = 0.1143

Cells in bold represent statistically significant differences.

Melatonin shows a daily rhythm in most species. However, in *C. sapidus* our results showed no statistical differences between time points or molt stages (Table 2) where the circulating levels of melatonin were evaluated (Figure 3B).

### 3.2 Relative Gene Expression of *CasEcR* and *CasMIH*

Besides analyzing the daily temporal expression of the ecdysteroid receptor and MIH genes at three-time points over 24 h, the study enabled the comparison of two molt stages, intermolt and premolt.

**FIGURE 4:**
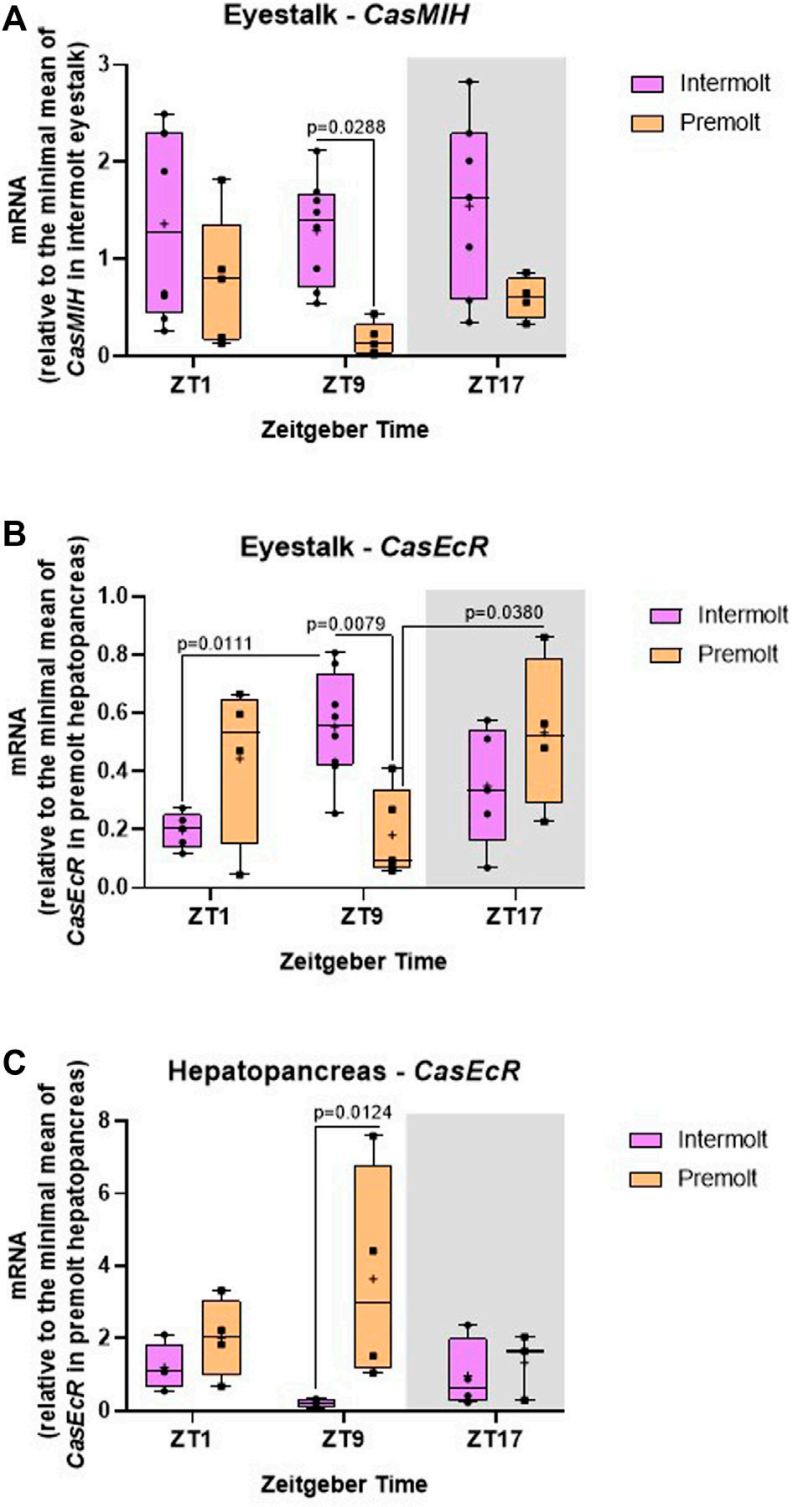
Temporal gene expression in intermolt and premolt *C. sapidus*. **(A)** *CasMIH* (*n* = 4–8) and **(B)** *CasEcR* (*n* = 4–8) in the eyestalks, and **(C)** *CasEcR* (*n* = 3–4) in the hepatopancreas. Total RNA was extracted 1, 9, and 17 h after lights on. Gene expression was normalized by *CasRPL12* and the values of *CasEcR* and *CasMIH* were expressed relative to the minimal mean of *CasEcR* in premolt hepatopancreas and of *CasMIH* in intermolt eyestalk, respectively. *p* values refer to the differences between molt stages, or temporal points within a single molt group, as determined by Two-way ANOVA, followed by Bonferroni post-test.

The relative *CasMIH* expression in the eyestalk did not vary among time points in any molt stage. The F value from ANOVA indicates a significant impact of the molt stage (Table 2) and was confirmed by the post-test (*p* = 0.0288) at ZT9 (Figure 4A).

We found a significant interaction between time and molt stage (Table 2) in *CasEcR* expression in the eyestalk. This gene exhibited a rhythm in both stages, with different patterns. Significant difference was observed at ZT9 between molt stages, with higher expression in the intermolt compared to premolt crabs (*p* = 0.079) (Figure 4B).

The hepatopancreas *CasEcR* showed a significant influence of the molt stage on its expression (Table 2). No variation among the time points was observed, however, a significant increase of *CasEcR* transcripts was seen at ZT9 in premolt compared to intermolt animals (*p* = 0.0124) (Figure 4C).

### 3.3 Effects of Exogenous Melatonin on the Gene Expression and Ecdysteroid Levels

Considering the possible effect of melatonin on the molt cycle, we analyzed the ecdysteroid levels and the relative expression of *CasEcR* and *CasMIH* genes in daily melatonin-injected intermolt crabs.

**FIGURE 5:**
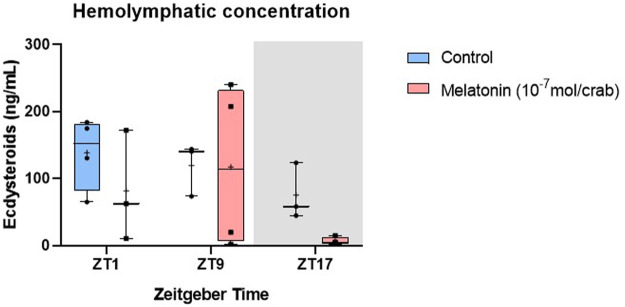
Concentration of ecdysteroids in intermolt *C. sapidus* hemolymph after daily injections of 10^−7^ mol melatonin/animal for 7 days. Ecdysteroids (*n* = 3–4) were extracted 1, 9 and 17 h after lights on. The values are expressed as median, quantiles, maximum, and minimum concentration.

No statistical differences were observed in the ecdysteroids levels between control and melatonin-treated animals (Figure 5). At ZT17 the ecdysteroid levels tend to decrease in the hormone-treated crabs, however, this reduction was not statistically significant (Table 3).

**TABLE 3:** ANOVA parameters related to gene expression and hormone concentrations in melatonin-injected crabs.

		Time		Melatonin treatment		Interaction	
		F (DFn,DFd)	*p* Value	F (DFn,DFd)	*p* Value	F (DFn,DFd)	*p* Value
Ecdysteroids	Hemolymph	(2, 15) = 2.466	*p* = 0.1186	(1, 15) = 1.862	*p* = 0.1925	(2, 15) = 0.4931	*p* = 0.6527
*CasMIH*	Eyestalk	(2, 19) = 2.250	*p* = 0.1327	(1, 19) = 16.40	***p* = 0.0007**	(2, 19) = 1.132	*p* = 0.3431
*CasECRI*	Hepatopancreas	(2, 23) = 5.190	***p* = 0.0138**	(1, 23) = 15.78	***p* = 0.0006**	(2, 23) = 2.483	*p* = 0.1055
		(2, 16) = 7.190	***p* = 0.0059**	(1, 16) = 2.209	*p* = 0.1566	(2, 16) = 19.53	***p* < 0.0001**

Cells in bold represents statistically differences.

**FIGURE 6:**
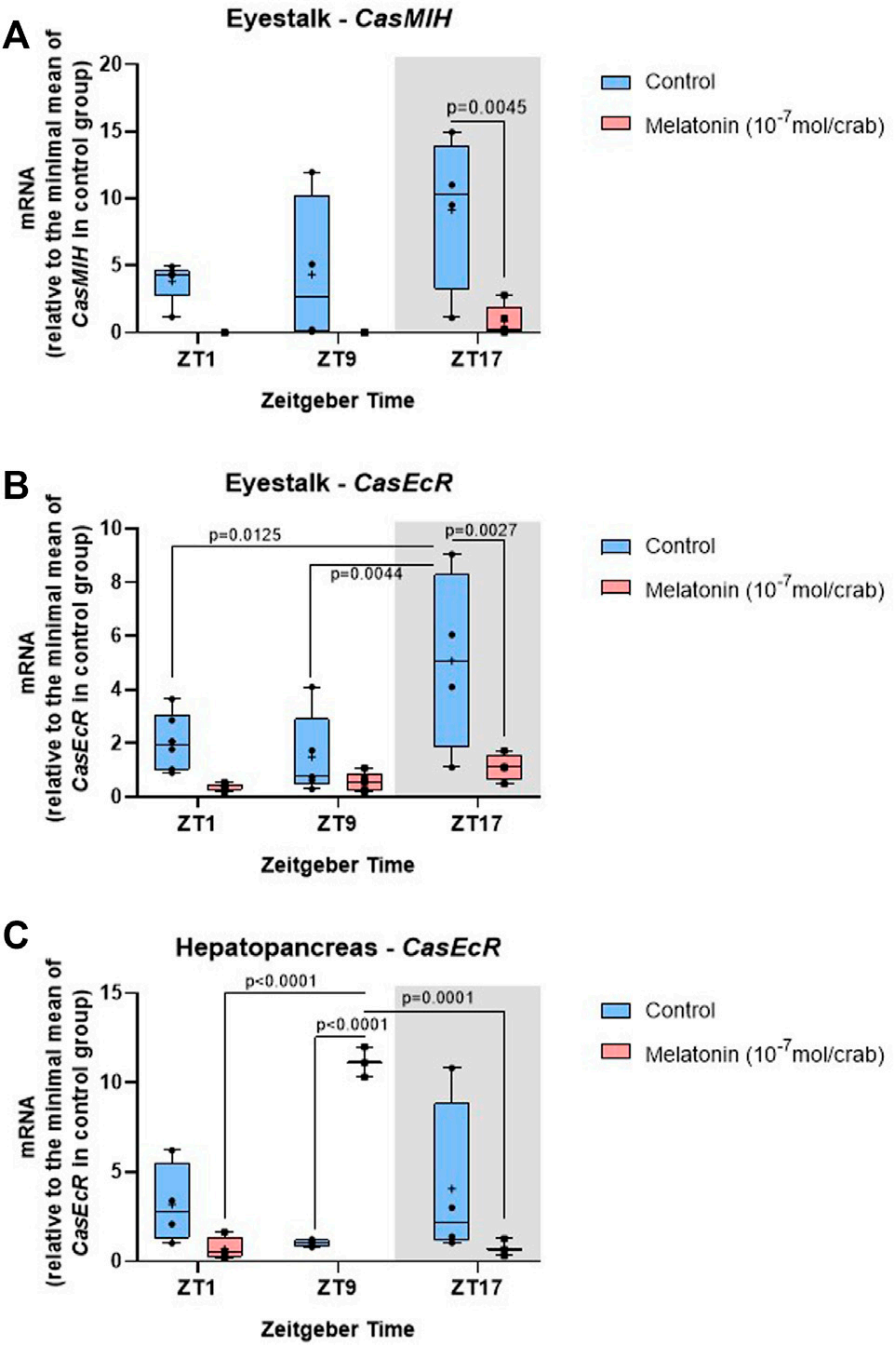
Temporal gene expression in intermolt *C. sapidus* after daily injections of 10^−7^ mol melatonin/animal for 7 days. **(A)** *CasMIH* (*n* = 3–5) and **(B)** *CasEcR* (*n* = 4–6) in the eyestalks, and **(C)** *CasEcR* (*n* = 3–4) in the hepatopancreas. Total RNA was extracted 1, 9, and 17 h after lights on. Gene expression was normalized by *CasRPL12,* and the values were expressed relative to the minimal mean of a control in each graph. *p* values refer to the differences between molt stages, or temporal points within a single molt group, as determined by Two-way ANOVA, followed by Bonferroni post-test.

In the eyestalk, the melatonin injection elicited a significant decrease in *CasMIH* expression (Table 3). This reduction was confirmed by the post-test at ZT17 (*p* = 0.0045) (Figure 6A). No oscillation in either molt group was found.

The F value indicates a significant impact of both factors, time and treatment in the eyestalk *CasEcR* expression (Table 3). Control animals showed an oscillatory profile with a peak of expression at ZT17, which was abolished in the melatonin-injected animals. At the same time point, the gene expression was reduced in melatonin-treated animals compared to the control crabs (*p* = 0.0027) (Figure 6B).

An interaction between time and melatonin treatment was observed in the hepatopancreas showing a significant influence on the *CasEcR* transcripts (Table 3). The treated group displayed an oscillation with a peak at ZT9 in comparison to ZT1 and ZT17. In addition, a significantly higher *CasEcR* expression at ZT9 in melatonin-treated animals than in the control group was observed (*p* < 0.0001) (Figure 6C).

## 4 Discussion

Reports about diurnal oscillation of molt cycle-related genes are extremely rare in decapod crustaceans. Because this is an overly complex physiological process, which comprises several stages and phases with a variety of hormones (Lipcius and Herrnkind, 1982; Lachaise et al., 1993; Chang, 1995; Nakatsuji et al., 2009; Chang and Mykles, 2011; Mykles, 2011; Webster et al., 2012), a thorough comprehensive analysis becomes exceedingly difficult. In addition, many environmental factors affect hormone synthesis and secretion, and gene expression, thus strongly influencing the molt cycle (Guo et al., 2013; Stoner et al., 2013; Gong et al., 2015).

Ecdysteroids, the main hormones responsible for ecdysis, are synthesized and secreted by the Y-organ, under the molt-inhibiting hormone negative regulation. The first aim addressed in this study was to evaluate ecdysteroid levels, ecdysteroid receptors, and the molt-inhibiting hormone expression, in two molt stages and at three time-points of the day, events not yet reported for *Callinectes sapidus.*

Due to the ecdysteroid ecdysis-inducing action, higher hormone levels are expected in premolt animals. Our data confirm the literature studies, which report that premolt animals have a higher concentration in comparison to intermolt animals at all evaluated ZTs (Lee et al., 1998; Nakatsuji et al., 2000; Nakatsuji and Sonobe, 2004; Techa and Chung, 2013). Moreover, a temporal variation in premolt crabs was noted with a reduction of the ecdysteroid levels at ZT17.

For gene expression, molt stage (eyestalk *CasMIH* and hepatopancreas *CasEcR*) or their interaction (eyestalk *CasEcR*) showed significant results. Eyestalk holds the visual ganglia and is responsible for the synthesis of circadian molecules such as melatonin in many crustaceans, for example, *Eriocheir sinensis* and *Palaemonetes sinensis* (Han et al., 2018), and *Neohelice granulata* (Maciel et al., 2008). The X-organ-sinus gland complex seems to be the master of this rhythmicity (Aréchiga et al., 1985; 1993) and it is under the direct influence of light (Glantz et al., 1983), which would impact the daily variation of the hormones and gene expression evaluated in this work. In fact, our results showed that *CasEcR* oscillates along the time in the eyestalk of intermolt and premolt animals, although with different patterns. On the other hand, *CasMIH* did not display the same profile. Bearing that in mind, one may suggest that the synthesis and release of some hormones exhibit a diurnal profile in the eyestalk, but not necessarily the expression of *CasMIH*. Besides that, the variation among individuals may mask a possible oscillatory profile.

The relative expression of *CasEcR* and *CasMIH* in the eyestalk shows a similar profile: Both genes displayed higher expression at ZT9 in the intermolt than in premolt crabs. Previous studies showed that ecdysteroid receptors and *CasMIH* gene are related in the eyestalk since EcR is a nuclear receptor, that possesses a binding site in the MIH promoter and then influences its expression.

The hepatopancreas is one of the main targets of ecdysteroids, whose levels are high in the premolt stage, preceding the ecdysis. This organ plays a particularly important role in metabolic regulation, and it is responsible for calcium storage, which is fundamental for a successful molt cycle (Becker et al., 1974; Zanotto and Wheatly, 2002). As feeding behavior changes along the molt cycle, it is expected that the organ’s cell composition undergoes modifications to meet the nutritional demand during the cycle (Lipcius and Herrnkind, 1982; Ortega et al., 2011). Increased *CasEcR* expression in the hepatopancreas has been reported in response to ecdysis induction (Girish et al., 2015).

In our results, there was an increase of hepatopancreas *CasEcR* transcripts in the premolt animals, which may be related to the receptor function as ecdysis comes closer and ecdysteroid concentration rises in the hemolymph. However, this increase was statistically significantly only at ZT9, suggesting a possible rhythm of the *CasEcR*. In *Rhodnius prolixus,* ecdysone receptors exhibit a circadian rhythm in specific tissues in response or anticipation of the daily peaks of the hormone (Vafopoulou and Steel, 2006). In *C. sapidus,* we demonstrated that the ecdysteroid levels were higher at ZT1 and ZT9, and the increase of *CasEcR* transcripts at ZT9 may be in response to an increase of circulating ecdysteroids.

Ecdysteroids, MIH, methyl farnesoate, and its inhibitor are classic regulators of the molt cycle (Borst et al., 2001; Nagaraju, 2007; 2011). A strong candidate as a positive factor is melatonin, whose functions in the vertebrates are linked to its pattern of synthesis and release in the dark (Carrilo-Vico et al., 2005; Markus and Ferreira, 2011; Cipolla-Neto et al., 2014) since the synthesis key enzyme is inhibited by light, even in nocturnal species (Reiter et al., 2014).

One point to highlight is that, unlike the majority of vertebrates, melatonin concentration peak in crustaceans may vary according to the species: during the photophase, as in *Procambarus clarkii* (Agapito et al., 1995), *Uca pugilator* (Tilden et al., 2001) and *Daphnia magna* (Markowska et al., 2009), or in both the photophase and the scotophase as in *N. granulata* (Maciel et al., 2008). In *C. sapidus*, circulating melatonin did not exhibit a temporal oscillation in intermolt or premolt crabs, and no concentration difference was seen between stages. However, preliminary data with more time points evaluated every 4 h, along the 24 h (data not shown) suggest that both, ecdysteroids and melatonin, display an increase at ZT9.

In crustaceans, light does not seem to affect melatonin synthesis: *C. sapidus* and other species presented the highest concentrations during the photophase, suggesting that factors other than light regulate melatonin production in these animals. Unfortunately, most reports about melatonin concentration in crustacean hemolymph did not mention the stage of the molt cycle, temperature, and light conditions, which may be the reason for the inconsistencies seen in the literature. In our study, we focused on two stages of the molt cycle, and kept constant light-dark cycle, temperature, and water conditions, to clarify the controversial data about melatonin rhythmicity in crustaceans. Nevertheless, further experiments should be performed to confirm this finding.

To analyze a possible positive effect of melatonin on the molt cycle, the intermolt crabs were daily injected with melatonin for 7 days at ZT6 and the ecdysteroid levels, ecdysteroid receptor, and MIH expression were determined. The reason to use intermolt crabs was to verify whether melatonin treatment would induce the ecdysis; the injection time was based on the majority of the literature reports which claim that melatonin peaks in the photophase.

The ecdysteroid levels did not show variation, but a reduction at ZT17 in melatonin-treated animals was visible; but probably due to the individual variability the difference was not statistically significant. In our hypothesis, melatonin should evoke the same effect as methyl farnesoate on the molt cycle (Reddy et al., 2004); in other words, we expected a melatonin-induced increase in the production and secretion of ecdysteroids. A possibility is that the melatonin injections were sufficient to induce a later stage of premolt, where the ecdysteroid levels were reduced in *C. sapidus*.

Our results revealed a similar expression pattern of both genes in the eyestalk, where exogenous melatonin inhibited their transcription at ZT17, whereas, in the hepatopancreas, melatonin increased *CasEcR* expression at ZT9, suggesting organ-specific melatonin receptors.

The eyestalk X-organ/sinus gland complex synthesizes and stores MIH, respectively, a hormone required during the later stages, posterior to ecdysis, the postmolt and intermolt stages (Philippen et al., 2000; Nakatsuji et al., 2009). Nevertheless, it has been reported that in some species MIH is synthesized in the premolt stage, stored in the sinus gland until the releasing time, but never with higher levels than in the postmolt and intermolt periods (Nakatsuji et al., 2000). One has also to consider the interaction between ecdysteroid receptor and MIH in the eyestalk, where *CasEcR* may be acting in the later premolt stage before ecdysis. Techa and Chung (2015) suggested that ecdysteroids exert positive feedback on the MIH expression: after reaching certain levels, the ecdysteroids inhibit the molt cycle via EcR stimulation of *MIH* transcripts. Bearing this in mind, one may suggest a positive action of melatonin on the molt cycle, in which the MIH inhibition may emanate from *CasEcR* inhibition. We hypothesized that melatonin acts as a positive regulator of the molt cycle through an inhibitory action on the molt inhibitor hormone in the eyestalk as discussed above.

Ecdysteroid receptors in the target organs, such as the hepatopancreas, display distinct functions from those in the eyestalk since the signaling in the hepatopancreas could induce ecdysis (Shechter et al., 2007; Huang et al., 2015). In fact, as to the hepatopancreas, melatonin-treated crabs, which were in the intermolt stage, showed an increase in *CasEcR* levels at ZT9; in addition, this gene exhibited an oscillation in treated animals. It would be expected that the expression of the ecdysteroid receptors increases with the melatonin treatment. Interestingly, melatonin-injected intermolt crabs showed *CasEcR* expression similar to premolt crabs (Figure 4C), corroborating the hypothesis that melatonin has a positive effect on the molt cycle.

Taken altogether, our results demonstrated the lack of temporal variation in *CasMIH* gene expression in premolt and intermolt eyestalk animals. On the other hand, *CasEcR* displayed an oscillatory profile with a peak at ZT9 in the eyestalk of intermolt animals; in the hepatopancreas, this gene seems to oscillate in premolt animals. Importantly, our study highlights important limitations in the literature regarding melatonin secretion rhythm in crustaceans. In *C. sapidus*, we are the first to demonstrate melatonin presence and how this hormone behaves in premolt and intermolt stages.

We also demonstrated that exogenous melatonin has a positive effect on the molt cycle, leading to a gene expression in intermolt animals’ pattern similar to the one found in premolt crabs, probably affecting eyestalk and hepatopancreas physiology. In the eyestalk, we found an indirect activation of the molt cycle (through *CasMIH* and *CasEcR* inhibition) whereas in the hepatopancreas the indoleamine increased the expression of *CasEcR*. Ultimately, our results widened the knowledge about the influence of melatonin on molt-related genes and daily hormone variation, bringing an essential contribution to the field of comparative endocrinology.

## Data Availability

The original contributions presented in the study are included in the article/Supplementary Materials, further inquiries can be directed to the corresponding author.
